# Sexual dimorphism in African elephant social rumbles

**DOI:** 10.1371/journal.pone.0177411

**Published:** 2017-05-10

**Authors:** Anton Baotic, Angela S. Stoeger

**Affiliations:** Mammal Communication Lab, Department of Cognitive Biology, University of Vienna, Vienna, Austria; Tierarztliche Hochschule Hannover, GERMANY

## Abstract

This study used the source and filter theory approach to analyse sex differences in the acoustic features of African elephant (*Loxodonta africana*) low-frequency rumbles produced in social contexts (‘social rumbles’). Permuted discriminant function analysis revealed that rumbles contain sufficient acoustic information to predict the sex of a vocalizing individual. Features primarily related to the vocalizer’s size, i.e. fundamental frequency variables and vocal tract resonant frequencies, differed significantly between the sexes. Yet, controlling for age and size effects, our results indicate that the pronounced sexual size dimorphism in African elephants *is partly*, *but not exclusively*, *responsible for* sexual differences in social rumbles. This provides a scientific foundation for future work investigating the perceptual and functional relevance of specific acoustic characteristics in African elephant vocal sexual communication.

## Background

Sexual dimorphism, describing morphological, physiological and behavioural differences of sexually mature females and males, is proposed to have evolved either through sexual selection or adaptations for sex-specific niche divergence [1]. In mammals with polygynous mating systems, selective processes producing sexual dimorphism result in different overall body size, with males being generally larger than females (male-biased size dimorphism) [2, 3]. Body size in animals is a crucial component in many aspects of ecology and social behaviour, and plays an important role in determining an individual’s fighting ability and reproductive success [2].

Studies on sound production and usage in various mammals showed that vocalizations encode honest information about a caller’s phenotype, including body size, age and sex (see [4] for review). Moreover, vocalizations play a key role for kin and individual recognition, e.g. in rhesus monkeys [5], wolves [6] and meerkats [7].

According to the source-filter theory, most mammal vocalizations are generated by the larynx and are subsequently filtered by the supra-laryngeal vocal tract (VT) [8]. The fundamental frequency (*F*0) of a vocalization is affected by the dimensions of the vocal folds (the source) [8, 9]. In nonhuman mammals, the exact underlying mechanisms of hormones in influencing vocal fold dynamics and acoustic structure have not been identified yet, but it has been experimentally shown that androgen stimulation has a direct effect upon the larynx [10]. Further studies demonstrated a correlation between androgen levels and the acoustic structure of vocalizations. For instance, higher androgen levels are correlated with higher-pitched solo songs in male gibbons [11] and with higher *F*0 modulation rates in male giant panda bleats [12]. Since testosterone is an important factor mediating competitive behaviours among males [13, 14], androgen-induced vocal cues may be generally important for intra-sexual assessment and mate preference.

As the source signal travels through the successive VT (the filter), its length and shape amplifies certain resonant frequencies (formants) selectively [8]: longer VTs produce lower, more closely spaced formants, reflecting larger body size [4]. This, together with the demonstration of formant perception by nonhuman mammals [15, 16], suggests that formants function as a universal cue to body size in terrestrial mammals [17]. In addition, intriguing sexually dimorphic adaptations–such as laryngeal air sacs in reindeer [18] a descended larynx in humans and red deer [19], an extremely enlarged larynx in hammerhead bats [20], or an additional set of vocal folds outside the larynx in koalas [21]–are important for mate selection and form the basis for most sex differences in animal vocalizations [4].

African elephants (*Loxodonta africana*) live in matrilineal societies in which most female vocalizations are used for family group and inter-individual cohesion [22]. Males leave their natal family at an average age of 14 years, but all-male coalitions and companionship have been reported [23]. Their vocalizations seem to be primarily related to contexts of intra-sexual dominance and reproduction [22]. Compared to females, however, little work has been done on male vocal behaviour in general, and studies addressing gender-dependent differences in vocalizations are mainly restricted to differences in vocal activity and vocalization types [22]. African elephants make use of 8–10 distinct call types [24–26], which grade with intermediate cross-forms between and within different call categories; this affects acoustic parameters including formants [27–31]. The most common call type is the “rumble”, a low-frequency and harmonically rich vocalization with frequencies in the infrasonic range, but with harmonics that extend well into the audible range [32]. Rumbles were suggested to have multiple social functions, from close-distance social interactions to reinforce social bonds [33] to the coordination of long-distance movements [34] and the maintenance of contact between spatially separated groups [35, 36].

Little is known about how elephants generate their distinct vocalizations, but recent experiments on low-frequency rumbles indicate flow-induced vocal fold vibration as the source for this particular call type [37].

Although elephants apparently follow the basic way of mammalian sound production, they possess an exceptionally elongated nasal VT (proboscis) compared to their oral VT [32]. Sound visualization experiments revealed that elephants control the vocal path from oral to nasal rumble production depending on context and thereby significantly vary the formant structure of their vocalizations [38].

Despite the extreme sexually dimorphic body size in African elephants [22], both sexes produce distinctive ‘sex-specific’ (i.e. produced by only one sex; in the manner of [39]) rumble types in reproductive contexts [34]. When mature males are in ‘musth’, a periodically occurring reproductive state characterized by aggressive behavior and highly elevated androgen levels [40], a distinctive pulsated ‘musth-rumble’ is emitted. Females in oestrus produce an ‘estrous-rumble’ [34]. It is, however, not acoustically distinctive and unique to the ovulatory follicular phase [25], which represents the second wave of follicular development that ultimately leads to ovulation [41]. Both rumble types are probably used to assess or advertise the behavioural and physiological state of/for a sexual partner and a sexual rival [32]. Whether sex differences in the acoustic structure are pronounced in functionally identical ‘social rumbles’, shared by both sexes, is currently unknown and requires clarification.

Cues about sex in social long-distance vocalizations might be particularly important in spatially and socially flexible species for mediating social dynamics. Elephant ranging patterns can cover huge areas, where hundreds of individuals of different age and sex could be encountered opportunistically [42]. Behavioural reactions depend upon the identity and maturity of encountered individuals [43, 44], but the influence of sex has not been explored. Here, we investigate whether source- and filter-related acoustic features encode sex-specific information in social rumbles and discuss their potential functional relevance for the African elephant’s vocal communication system.

## Material and methods

### Study subjects and housing

Our study subjects were 9 adult female and 10 adult male elephants from two European zoos and four South African privately owned elephant keeping reserves (Table 1). All elephants have social contact throughout the day and spend the night in separated stables (but do have tactile, visual or acoustic contact). Elephants housed in South Africa were allowed to free-roam in areas of 3–45 km^2^ for several hours per day.

**Table 1:** Study sites, sex, age and number of calls for each study subject.

Sex	Location, year of data collection	Individual	Origin	Age (years)	approx. shoulder height (cm)	N calls (used in analysis)
**Males**	Addo Elephant Back Safaris, 2015	Duma	Kruger National Park, South Africa	∼28	325	14
	Pilanesberg, 2014	Mana	Mana Pools National Park, Zimbabwe	∼29	325	13
		Mike	Mana Pools National Park, Zimbabwe	∼29	320	17
		Sapi	Mana Pools National Park, Zimbabwe	∼30	325	10
	Bela Bela, 2014	Chishuru	Limpopo, South Africa	∼19	240	13
		Chova	Limpopo, South Africa	∼21	250	18
	Hazyview, 2014	Medwa	Limpopo, South Africa	∼19	260	11
		Shamwari	Limpopo, South Africa	∼19	270	10
		Tembo	Kruger National Park, South Africa	∼34	330	20
		Ziziphus	Limpopo, South Africa	∼18	250	14
**Females**	Vienna Zoo, 2016	Mongu	Vienna Zoo	13	230	14
		Tonga	Kruger National Park, South Africa	31	250	20
		Drumbo	Zimbabwe	41	240	10
		Numbi	Kruger National Park, South Africa	24	230	20
	Tierpark Berlin, 2016	Pori	Hwange National Park, Zimbabwe	35	250	4
	Bela Bela, 2014	Mussina	Limpopo, South Africa	12	220	13
		Nuanedi	Limpopo, South Africa	13	210	12
		Shan	Limpopo, South Africa	∼16	230	15
	Pilanesberg, 2014	Chikwenya	Mana Pools National Park, Zimbabwe	∼29	240	15

tilde (∼) indicates that the exact birth date is unknown.

### Data collection

Members of elephant herds mostly move together, accompanied by brief periods of spatial and temporal separation of individuals for foraging [36]. Rumbles mediate spatial relationships within a herd and thus promote group cohesion [45], even when out of visual range [35]. To allocate social rumbles to the vocalizing elephant, we focused on particular individuals during the following recording situations: (1) feeding: where the focus individual performs exclusively browsing and feeding activities (S1 Video), either within visual range or when segregated from its group members; (2) active approach: rumbling when approaching another group member; (3) passive approach: rumbling when being approached by another group member (S2 Video); (4) in reaction to physical contact: for example, in reaction to a trunk-touch (no agonistic interactions such as pushing or tusking); (5) general locomotion of the group: physical movement of all group members in the same direction, as for example when leaving enclosures together; and (6) spatial separation: vocalizing elephant is spatially separated from the rest of the group due to the daily routine of the respective institution (S3 Video, S1 Table).

We recorded throughout the day between 7 a.m. and 5 p.m. Recordings at both European zoos were made from protected keeper areas 5–20 m from the elephants. In South Africa we followed the animals on foot accompanied by elephant handlers for security reasons, did not interact with the animals, and passively recorded at distances ranging from 5–50 m.

The elephants’ individual identification was based on their physical characteristics, such as body size, genitals, shape and patterns of notches and holes in the ears, length and shape of tusks and tail, and the presence of specific marks on the body (e.g. warts). Moreover, since African elephants often raise and flap their ears while rumbling and, as if listening, sometimes hold very still with their head and ears lifted before or after vocalizing [34] (see S1, S2 and S3 Videos for demonstration), we additionally used behavioural categories such as ear and head movement to identify the calling individual. The identity of the focus elephant was, however, noted only when both authors agreed upon the vocalizing individual. No behavioural or visual signals of oestrus and musth occurred [46].

Shoulder height of the study subjects was measured as the vertical distance from the ground up to the top of the shoulder. Individuals from South Africa were measured using a Telefix 4 telescopic meter. Measurements at Vienna Zoo and Tierpark Berlin were taken by the keepers using a measuring scale attached along the steel-bar of the enclosures. The respective institution provided information about age for each individual.

Acoustic data were obtained using an omni-directional Neumann KM183 condenser microphone, modified for recording frequencies below 20 Hz (flat-recording down to 5 Hz), connected to a Sound Devices 722 (frequency response: 10 Hz–40 kHz) at 48 kHz sampling rate and 16-bit.

### Acoustic analysis

Data were annotated in S_Tools-STx 4.2.8 [47] by defining the on- and off-set of each elephant rumble. Prior to in-depth acoustic analysis, we pre-selected calls with specific formant locations, namely formant 1 (F1) and 2 (F2), potentially indicating nasally emitted rumbles. Using an estimated VT length of 0.75 m for oral and 2.5 m for nasal rumbles, the predicted formant locations for oral rumbles are F1 = 116.7 Hz, F2 = 350.0 Hz, and for nasal rumbles F1 = 35.0 Hz, F2 = 105.0 Hz, respectively [32]. Therefore, we classified rumbles as nasally produced if F1 was below 50 Hz and F2 below 150 Hz. We then used a customized semi-automatic Matlab sound analysis tool [48] to extract source-related acoustic features by tracing the *F*0-contour in Fast Fourier spectrograms (frame size: 300 ms, step size: 40 ms). To analyse filter-related formants, we used linear predictive coding (LPC) in S_Tools-STx to extract frequency values of the first two formants. Definitions of the source- and filter-related acoustic parameters are provided in S2 Table.

### Statistical analysis

Our selections yielded 140 rumbles from 10 males and 123 rumbles from 9 female elephants (S1 Table) recorded in different sessions over an overall data collection time of 50 days (mean 7.2 ± 2.9 days per elephant, range 3–17 days).

An initial multivariate analysis of variance (MANOVA) was carried out to identify significantly different acoustic parameters between sexes. To correct for the effects of multiple testing and the occurrence of false positives, all P-values were adjusted using the Benjamini-Hochberg False Discovery Rate (BH FDR) procedure [49]. Acoustic features above the BH FDR corrected significance level were discarded as insignificant and not used for further analysis. To evaluate whether the data were suitable for conducting a principal component analysis (PCA), we used the Kaiser-Meyer-Olkin (KMO) measure of sampling adequacy and performed the Bartlett’s test of Sphericity. The verified acoustic parameters were then entered into a varimax rotated PCA with Kaiser-normalization for data reduction. Resulting factor scores with eigenvalues greater than one were retained, saved using the regression method and utilized as input for classification analysis. To test the classification of individual elephant rumbles to sex, we performed a permuted discriminant function analysis (pDFA), a randomization procedure used for non-independent two-factorial data sets when one factor is nested in another [50]. The pDFA was conducted using a function (provided by R. Mundry) based on Ida of the R package MASS [51]. We defined sex as test and individuality as control factor. Although age and shoulder height are tightly correlated in both sexes of African elephants [52], we controlled for the effects of both age and size in males (Pearson Correlation, r = 0.944, p < 0.001) and females (Pearson Correlation, r = 0.747, p < 0.001) by conducting 2 separate pDFAs. Five age groups were established (S3 Table) and entered as restriction factor to control for age effects. The second pDFA used 5 shoulder height categories as restriction factor to control for the effect of size (S4 Table). Further, to evaluate the influence of source-related parameters only, we repeated all statistical procedures by excluding filter-related variables. Results are expressed as percentage of correct classification (cross-validated). All statistical tests were performed in SPSS v.22 and RStudio v.1.0.136.

## Results

Using the BH FDR corrected significance level (q* = 0.0296), the initial MANOVA verified significant differences in 16 variables: in all filter-related parameters, all absolute frequency parameters (except ‘Min *F*0’ and ‘Max *F*0/Mean *F*0’), and 2 shape- and contour-related parameters (‘coefficient of frequency modulation’ (COFM) and ‘jitter factor’). None of the temporal-related parameters were significantly discriminant between sexes (Table 2). The subsequent PCA was justified as shown by the KMO criterion (0.802) and the Bartlett’s test of Sphericity (*X*^2^ = 16,876.96, *df* = 120, *p* < 0.001).

**Table 2:** MANOVA comparing source- and filter-related acoustic features between male and female African elephant social rumbles.

	Manova			Mean ± SD	
Source-related parameters	F	df	*P*	Males	Females
**Absolute frequency parameters (Hz)**					
Start *F*0	6.861	1	0.009	12.21 ± 2.22	12.94 ± 2.27
Middle *F*0	37.178	1	<0.001	14.35 ± 2.35	16.21 ± 2.61
Finish *F*0	5.596	1	0.019	11.85 ± 2.32	12.51 ± 2.23
Min *F*0	4.462	1	0.036*	11.16 ± 2.05	11.67 ± 1.75
Max *F*0	41.381	1	<0.001	15.27 ± 2.29	17.16 ± 2.48
Range *F*0	33.088	1	<0.001	4.10 ± 1.48	5.49 ± 2.38
Mean *F*0	41.471	1	<0.001	13.46 ± 2.01	15.09 ± 2.09
Median *F*0	40.696	1	<0.001	13.64 ± 2.07	15.35 ± 2.27
Mean 1^st^ Third	39.786	1	<0.001	13.25 ± 2.01	14.81 ± 2.01
Mean 2^nd^ Third	41.245	1	<0.001	14.22 ± 2.18	16.06 ± 2.48
Mean 3^rd^ Third	30.152	1	<0.001	12.90 ± 2.24	14.38 ± 2.10
Max *F*0/Mean *F*0	0.088	1	0.767*	1.14 ± 0.05	1.14 ± 0.04
Mean *F*0/Min *F*0	22.051	1	<0.001	1.22 ± 0.11	1.31 ± 0.21
**Temporal parameters (s)**					
Duration	2.883	1	0.091*	3.8857 ± 2.2276	4.2725 ± 1.2712
Min *F*0 Location	0.330	1	0.566*	0.5231 ± 0.4764	0.4894 ± 0.474
Max *F*0 Location	1.819	1	0.179*	0.3738 ± 0.2603	0.336 ± 0.1818
Time Minimum to Maximum	2.558	1	0.111*	0.5798 ± 0.2508	0.5327 ± 0.2234
**Shape and contour parameters**					
Coefficient of Frequency Modulation (COFM) [53]	123.735	1	<0.001	0.0009 ± 0.0005	0.0018 ± 0.0009
Jitter Factor [54]	74.958	1	<0.001	7.4005 ± 2.4982	4.4991 ± 2.9361
Frequency Variability Index [54]	0.668	1	0.415*	0.1109 ± 0.0706	0.1039 ± 0.0679
Inflection Factor	0.332	1	0.565*	0.3989 ± 0.1576	0.3889 ± 0.1179
Start Slope	0.592	1	0.442*	2.2721 ± 4.3308	2.6155 ± 2.5546
Middle Slope	0.911	1	0.341*	0.0345 ± 2.8188	-0.2186 ± 0.8937
Final Slope	0.473	1	0.492*	-2.029 ± 2.5882	-2.2193 ± 1.7562
**Filter-related parameters (Hz)**					
Formant 1	78.357	1	<0.001	24.81 ± 4.12	30.35 ± 5.48
Formant 2	107.305	1	<0.001	89.93 ± 16.42	110.53 ± 12.91
Formant Dispersion	69.883	1	<0.001	65.12 ± 14.04	80.18 ± 12.55

Acoustic features above the Benjamini-Hochberg corrected significance level (q* = 0.0296) were treated as insignificant and discarded from principal component analysis.

The PCA reduced 16 significant source- and filter-related parameters to 3 principal components (PC) explaining 82.5% of the total variation: most source-related parameters correlated strongly with PC 1 explaining 45.6% of the variance. Shape- and contour-related variables were assigned to PC 2 explaining 21.6% of the variance, whereas filter-related parameters strongly correlated to PC 3 explaining 15.3% of the variance. Loading values for each parameter that loaded strongly to one of the three PC are shown in S5 Table.

The pDFA, controlling for an effect of age, resulted in 85.6% correct cross-validated classification after 100 random selections and 10,000 permutations. This shows that female and male elephants are discriminable based on 16 acoustic parameters of social rumble vocalizations (*p* = 0.0207). The second pDFA, comparing both sexes using ‘shoulder height’ as restriction factor, resulted in 85.6% correct cross-validated classification (*p* = 0.0187).

After excluding the filter-related variables, a second MANOVA identified the same 13 source-related parameters (*F*, *df* and *p* values are identical as in Table 2) as being significantly different (q* = 0.0271) between the sexes. The subsequent second PCA was justified by the KMO measure of sampling adequacy (0.798) and the Bartlett’s test of Sphericity (*X*^2^ = 7,284.059, *df* = 78, *p* < 0.001). The 13 variables were then reduced to 2 PCs explaining 80.9% of the total variation (loading values for each parameter are shown in S6 Table). Controlling for the effect of age resulted in 73.2% (*p* = 0.0367) correct cross-validated classification. When controlling for size effects, a correct cross-validated classification of 72.9% (*p* = 0.1297) was achieved.

**Fig 1:**
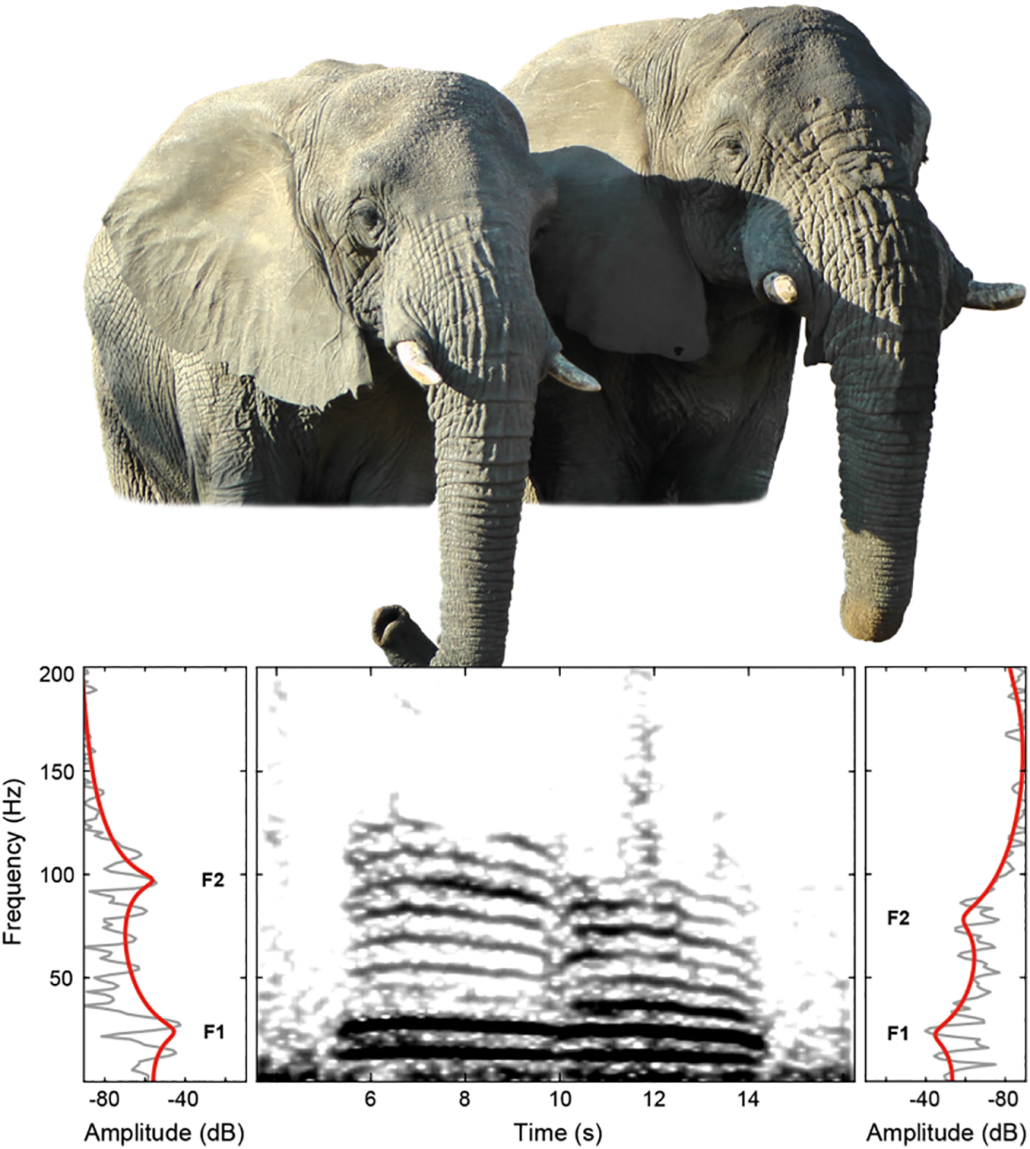
Comparison of a female (left, Chikwenya) and a male (right, Mike) African elephant. The spectrogram and power spectra below the photograph provide an example of a social rumble of each sex, indicating formant positions (rumbles uttered first by Chikwenya, followed by Mike).

Fig 1 illustrates comparative spectrograms of a female and male elephant social rumble. The corresponding acoustic recording is provided in S1 Sound, with the female rumbling first.

## Discussion

This study presents the first comparative acoustic analysis of adult female and male African elephant vocalizations. It considers source- and filter-related acoustic features of low-frequency rumbles emitted in non-reproductive social contexts. Our results demonstrate that female and male social rumbles encode reliable information about the physical attributes and sex of the caller.

Elephant vocal folds are voluminous and long, reaching about 10 cm in adult females [37]. The dimensions of the vocal folds determine the vibrational behaviour during phonation and hence determine the *F*0 [9, 17]. When excluding formants (which are known reliable indicators for body size), source-related features, i.e. absolute frequency, shape and contour parameters, showed a considerable and significant classification success between the sexes when controlling for age, but a non-significant result controlling for size. Due to the size dimorphism, in our data set the same individuals assigned to their respective age category are not necessarily found in the same shoulder height category. This non-significant p-value could reflect the small sample size in some size categories. Notwithstanding, this might mean that most of our analysed source-related parameters are influenced by size effects. Our findings go along with previous studies showing that source-related vocal features reflect size-related differences between age categories in many species (e.g., baboons [55] and goats [56]), between sexes (baboons [57]) and within species exhibiting a large morphological range (domestic dogs [58]). Beyond size, increased male androgen levels might affect fundamental frequency. Vocal folds are highly sensitive to testosterone [10], leading to lower *F*0 in human males (by potentially changing vocal fold bulk, length or tension of the vocal folds) [59]. Accordingly, increased levels of circulating testosterone affecting vocal fold dynamics might further cause a sexual difference of the *F*0 in elephant social rumbles. Also, COFM and jitter factor–acoustic features representing the shape and contour features of the F0 –differed significantly between the sexes. Jitter, typically an index for *F*0-variability, was higher in male elephants than in females. Females, however, had higher COFM values, indicating high frequency modulation [60], and thus more modulated rumbles than those of males. A similar result was observed in giant pandas, where circulating oestrogens and testosterone were suggested to affect vocal fold oscillations in each sex [61]. Elephant larynx anatomy differs proportionally in size and structure compared to humans, and a highly complex vocal fold vibratory pattern, previously not documented in other species, has been reported [37]. This complicates data interpretation of *F*0 shape and contour features. Whether these parameters are related to testosterone levels [61] requires anatomical and physiological investigations.

Elephants are among the most size dimorphic of all mammals: males can be twice the mass of a female. Also, secondary sexual characteristics between both sexes such as dimorphism of tusk and skull size exist [22]. Particularly, skull and mandible size are larger and more pronounced in male elephants, including more robust muscle markings and attachments [62]. The most rostral part of the skull is formed by the paired incisive bones, *Os incisivum* (located at the tip of the maxilla), which are more developed and rostrally expanded in bulls [63]. Nonetheless, it remains to be determined whether a morphological dimorphism in the vocal apparatus (e.g. in the howler monkey [64] and the Mongolian gazelle [65]) occurs other than size-related differences. In elephants it is argued that pharyngeal pouches present in both sexes might have an effect on the vocal output [66]. The acoustic role and method of sound production for these anatomical structures remain unexplored in both sexes.

Note also that social rumbles were recorded from individuals that were either born in captivity, wild-born orphans but raised in captivity from a young age on, or wild-caught as adults. Ultimately, (apart from captive-borns) all originate from different regions in Africa, where elephants occur in varying sizes across the continent [52, 67]. This inter-population variability in body sizes or stature has been suggested to be influenced by resource availability in the elephants’ environment and as a result of genetic variation because some populations differ considerably in certain haplotypes [52]. Our elephants had access exclusively to a highly nutritious diet, which in combination with little physical activity (some of our zoo elephants) leads to faster growth rates and higher weight compared to their wild counterparts [68]. We therefore emphasize that different life histories and life styles of study animals, and inter-population variances in certain demographic and morphometric parameters, should not be disregarded because they may play a relevant role for data interpretation.

Apart from comparing the acoustic structure between different cycle stages in females, no correlational studies have been conducted between hormone levels and acoustic parameters of these two rumble types. This calls for future research correlating vocalizations with sex steroid levels in both sexes to better understand the effect of testosterone. Most interestingly, this includes the transition to and/or the onset of the distinctive ‘musth-rumble’ during periods of extremely elevated androgen levels in males [69].

In conclusion, our results provide a clear distinction between male and female African elephant social rumbles. This is evidence that certain acoustic features facilitate the recognition of sex and are likely to be important for elephant social dynamics and inter-sexual vocal communication, which is, so far, poorly understood. In addition, interest in automatic acoustic monitoring of elephant populations is increasing [70–72], and early warning systems for people living in human-elephant conflict areas have become an issue [48, 73]. Vocal cues can be used as indicators of group size [70] and to categorize age groups based on vocalizations. This has considerable potential to help assess the demography of a monitored population [74]. Information on acoustic cues that reliably categorize sex could optimise algorithm development and make acoustic monitoring and detection systems more sophisticated. Accordingly, deterrent methods could be adjusted depending on the sex and the age of approaching elephants (e.g. a female group with infants versus males).

Finally, this study provides the framework for future research to further assess the role of particular acoustic features for inter- as well as for intra-sexual elephant communication by using, for example, re-synthesis techniques in acoustic playback experiments.

## Ethics statement

This non-experimental research meets all applicable European Union and South African laws and was conducted din accordance with the Guidelines for the Treatment of Animals in Behavioural Research and Teaching [75]. All participating institutions mentioned in this manuscript approved data collection for the study. The nature of the study was purely observational: No invasive methodologies were applied at any point of the study. The research did not affect the housing, daily routine, behaviour, diet or management of the animals. Therefore, no ethics committee approval was required.

## Supporting information

S1 Video Male African elephant rumbling during browsing and feeding activity. (MP4)

S2 Video Male African elephant rumbling while being approached. (MP4)

S3 Video Male African elephant rumbling in a spatial separation situation. (MP4)

S1 Table Dataset. (XLSX)

S2 Table Description of the source- and filter-related parameters measured. (DOCX)

S3 Table Age classification. (DOCX)

S4 Table Shoulder height classification. (DOCX)

S5 Table Extracted factors of the principal component analysis. (DOCX)

S6 Table Extracted factors of the principal component analysis. (DOCX)

S1 Sound Sound sample of a low-arousal social rumble of each sex, with the female rumbling first. Maximum frequency of the sound file was set to 200 Hz and the absolute peak of the amplitude was scaled to 0.99.
(WAV)
