# Supplementary material for: Sexual dimorphism in African elephant social rumbles
Source: PLoS One. 2017 May 10;12(5):e0177411. doi: 10.1371/journal.pone.0177411 (PMC5425207; doi:10.1371/journal.pone.0177411)
Supplement: S2 Table — (DOCX) [file pone.0177411.s005.docx]

**S2 Table. Description of the source- and filter-related acoustic parameters measured.**

| **Acoustic parameter** | **Description** |
| --- | --- |
| **Source-related parameters** |  |
| **Absolute frequency parameters** |  |
| Start F0 | Fundamental frequency at the onset of a rumble (Hz) |
| Middle F0 | Frequency at the temporal middle of a rumble (Hz) |
| Finish F0 | Fundamental frequency at the end of a rumble (Hz) |
| Min F0 | Lowest measured frequency of the fundamental (Hz) |
| Max F0 | Highest measured frequency of the fundamental (Hz) |
| Range F0 | Maximal fundamental frequency minus minimum fundamental frequency (Hz) |
| Mean F0 | Calculated as average frequency across the fundamental (Hz) |
| Median F0 | Median of all the frequencies of the measured harmonic (Hz) |
| Mean 1st Third | Mean frequency of the first third of a rumble (Hz) |
| Mean 2nd Third | Mean frequency of the second third of a rumble (Hz) |
| Mean 3rd Third | Mean frequency of the third of a rumble (Hz) |
| Max F0/Mean F0 | Calculated as peak frequency divided by mean frequency (Hz) |
| Mean F0/Min F0 | Calculated as mean frequency divided by minimum frequency (Hz) |
|  | |
| **Temporal parameters** | |
| Duration | Temporal distance of rumble measured in seconds (s) |
| Min F0 Location | Location of the minimum frequency on the fundamental contour (s) |
| Max F0 Location | Location of the maximum frequency on the fundamental contour (s) |
| Time Minimum to Maximum | Temporal distance from the minimum frequency to the maximum frequency (s) |
|  | |
| **Shape and contour parameters** | |
| Coefficient of Frequency Modulation (COFM)^1^ | Calculated variable that represents the amount and magnitude of frequency modulation across a rumble, computed by summing the absolute values of the difference between sequential frequencies divided by 10,000. |
| Jitter Factor^2^ | Calculated variable that represents a weighted measure of the amount of frequency modulation, by calculating the sum of the absolute value of the difference between two sequential frequencies divided by the mean frequency. The sum result is then divided by the total number of points measured minus 1 and the final value is obtained by multiplying it by 100. |
| Frequency Variability Index^2^ | Calculated variable that represents the magnitude of frequency modulation across a rumble, computed by dividing the variance in frequency by the square of the average frequency of a rumble and then multiplying the value by 10. |
| Inflection Factor | Percentage of points showing a reversal in slope |
| Start Slope | Calculated as (frequency 20-frequency 1)/(Time 20-Time 1) |
| Middle Slope | Calculated as (frequency 40-frequency 20)/(Time 40-Time 20) |
| Final Slope | Calculated as (frequency 60-frequency 40)/(Time 60-Time 40) |
|  |  |
| **Filter-related parameters** | |
| Formant 1 | First spectral peak of the LPC smoothed spectrum in the range of 0 to 200 Hz (model order 8) |
| Formant 2 | Second spectral peak of the LPC smoothed spectrum in the range of 0 to 200 Hz (model order 8) |
| Formant Dispersion | 2^nd^ formant minus 1^st^ formant |

1. McCowan B, Reiss D. Whistle contour development in captive-born infant bottlenose dolphins (*Tursiops truncatus*): Role of learning. Journal of Comparative Psychology. 1995;109(3):242-60. Doi: 10.1037/0735-7036.109.3.242
2. Mitani JC, Brandt KL. Social Factors Influence the Acoustic Variability in the Long-distance Calls of Male Chimpanzees. Ethology. 1994;96(3):233-52. doi: 10.1111/j.1439-0310.1994.tb01012.x.
