# Supplementary material for: Sexual dimorphism in African elephant social rumbles
Source: PLoS One. 2017 May 10;12(5):e0177411. doi: 10.1371/journal.pone.0177411 (PMC5425207; doi:10.1371/journal.pone.0177411)
Supplement: S3 Table — (DOCX) [file pone.0177411.s006.docx]

| **Age group (years)** | | | | | | | | | |
| --- | --- | --- | --- | --- | --- | --- | --- | --- | --- |
| **12−16**  **(n = 4)** | | **18−19**  **(n = 4)** | | **21−24**  **(n = 2)** | | **28−30**  **(n = 5)** | | **31-41**  **(n = 4)** | |
| Mussina | 12, f | Zizipuhs | 18, m | Chova | 21, m | Duma | 28, m | Tonga | 31, f |
| Nuanedi | 13, f | Chichuru | 19, m | Numbi | 24, f | Chikwenya | 29, f | Tembo | 34, m |
| Mongu | 13, f | Medwa | 19, m |  |  | Mana | 29, m | Pori | 35, f |
| Shan | 16, f | Shamwari | 19, m |  |  | Mike | 29, m | Drumbo | 41, f |
|  |  |  |  |  |  | Sapi | 30, m |  |  |

**S3 Table. Classification of African elephants by age.** Name, age (years) and sex (f = female, m = male) for each individual are given for each age group.
