# Supplementary material for: Sexual dimorphism in African elephant social rumbles
Source: PLoS One. 2017 May 10;12(5):e0177411. doi: 10.1371/journal.pone.0177411 (PMC5425207; doi:10.1371/journal.pone.0177411)
Supplement: S4 Table — (DOCX) [file pone.0177411.s007.docx]

| **Shoulder height group (cm)** | | | | | | | | | |
| --- | --- | --- | --- | --- | --- | --- | --- | --- | --- |
| **210−230**  **(n = 5)** | | **240**  **(n = 3)** | | **250**  **(n = 4)** | | **260−270**  **(n = 2)** | | **320-330**  **(n = 5)** | |
| Nuanedi | 210, f | Drumbo | 240, f | Tonga | 250, f | Medwa | 260, m | Mike | 320, m |
| Mussina | 220, f | Chichuru | 240, m | Pori | 250, f | Shamwari | 270, m | Duma | 325, m |
| Numbi | 230, f | Chikwenya | 240, f | Chova | 250, m |  |  | Mana | 325, m |
| Mongu | 230, f |  |  | Ziziphus | 250, m |  |  | Sapi | 325, m |
| Shan | 230, f |  |  |  |  |  |  | Tembo | 330, m |

**S4 Table. Classification of African elephants by shoulder height.** Name, shoulder height (cm) and sex (f = female, m = male) for each individual are given for each age shoulder height group.
