# Supplementary material for: Sexual dimorphism in African elephant social rumbles
Source: PLoS One. 2017 May 10;12(5):e0177411. doi: 10.1371/journal.pone.0177411 (PMC5425207; doi:10.1371/journal.pone.0177411)
Supplement: S5 Table — (DOCX) [file pone.0177411.s008.docx]

**S5 Table. Percentage of total variation and vector loading values of the source- and filter-related parameters contributing to sexual dimorphism in African elephant social rumbles.**

|  | **Principal Component** | | |
| --- | --- | --- | --- |
| **Acoustic features** | **1** | **2** | **3** |
| **Source-related parameters** |  |  |  |
| **Absolute frequency parameters** |  |  |  |
| Finish F0 | **0.837** | -0.224 | 0.187 |
| Max F0 | **0.881** | 0.419 | 0.124 |
| Mean F0 | **0.930** | 0.305 | 0.184 |
| Range F0 | 0.311 | **0.897** | -0.139 |
| Mean F0/Min F0 | 0.008 | **0.930** | -0.154 |
| Start F0 | **0.705** | -0.212 | 0.291 |
| Middle F0 | **0.849** | 0.422 | 0.097 |
| Mean 1st Third | **0.865** | 0.236 | 0.261 |
| Mean 2nd Third | **0.877** | 0.417 | 0.104 |
| Mean 3rd Third | **0.909** | 0.201 | 0.167 |
| Median F0 | **0.905** | 0.359 | 0.153 |
|  |  |  |  |
| **Shape and contour parameters** |  |  |  |
| Coefficient of Frequency Modulation (COFM) | 0.239 | **0.739** | 0.292 |
| Jitter Factor | -0.123 | **-0.366** | -0.241 |
|  |  |  |  |
| **Filter-related parameters** |  |  |  |
| Formant1 | **0.546** | 0.396 | 0.440 |
| Formant2 | 0.298 | 0.084 | **0.933** |
| Formant Dispersion | 0.154 | -0.044 | **0.942** |
|  | | | |
| **Rotation Sums of Squared Loadings** |  |  |  |
| Total | 7.30 | 3.46 | 2.45 |
| Percentage of variance | 45.64 | 21.60 | 15.30 |
| Percentage of cumulative variance | 45.64 | 67.24 | 82.54 |

Rotation Method: varimax with Kaiser Normalization. Vector loading values of parameters that loaded strongly to one of the three principal components are bold-typed.
