# Supplementary material for: Sexual dimorphism in African elephant social rumbles
Source: PLoS One. 2017 May 10;12(5):e0177411. doi: 10.1371/journal.pone.0177411 (PMC5425207; doi:10.1371/journal.pone.0177411)
Supplement: S6 Table — (DOCX) [file pone.0177411.s009.docx]

**S6 Table. Percentage of total variation and vector loading values of source-related parameters only contributing to sexual dimorphism in African elephant social rumbles.**

|  | **Principal Component** | |
| --- | --- | --- |
| **Acoustic features** | **1** | **2** |
| **Source-related parameters** |  |  |
| **Absolute frequency parameters** |  |  |
| Finish F0 | **0.871** | -0.145 |
| Max F0 | **0.843** | 0.502 |
| Mean F0 | **0.918** | 0.386 |
| Range F0 | 0.185 | **0.939** |
| Mean F0/Min F0 | -0.109 | **0.941** |
| Start F0 | **0.782** | -0.167 |
| Middle F0 | **0.804** | 0.504 |
| Mean 1st Third | **0.888** | 0.299 |
| Mean 2nd Third | **0.835** | 0.500 |
| Mean 3rd Third | **0.900** | 0.283 |
| Median F0 | **0.882** | 0.439 |
|  |  |  |
| **Shape and contour parameters** |  |  |
| Coefficient of Frequency Modulation (COFM) | 0.255 | **0.733** |
| Jitter Factor | -0.182 | **-0.328** |
|  |  |  |
| **Rotation Sums of Squared Loadings** |  |  |
| Total | 6.79 | 3.73 |
| Percentage of variance | 52.21 | 28.67 |
| Percentage of cumulative variance | 52.21 | 80.88 |

Rotation Method: varimax with Kaiser Normalization. Vector loading values of parameters that loaded strongly to one of the three principal components are bold-typed.
